# Shift of Circadian Feeding Pattern by High-Fat Diets Is Coincident with Reward Deficits in Obese Mice

**DOI:** 10.1371/journal.pone.0036139

**Published:** 2012-05-03

**Authors:** Lidia Morales, Nuria Del Olmo, Ismael Valladolid-Acebes, Alberto Fole, Victoria Cano, Beatriz Merino, Paula Stucchi, Daniela Ruggieri, Laura López, Luis Fernando Alguacil, Mariano Ruiz-Gayo

**Affiliations:** 1 Departamento de Ciencias Farmacéuticas y de la Alimentación, Facultad de Farmacia, Universidad CEU-San Pablo, Madrid, Spain; 2 Instituto Pluridisciplinar, Universidad Complutense, Madrid, Spain; 3 Unidad Traslacional, Hospital General de Ciudad Real, Ciudad Real, Spain; Sapienza University of Rome, Italy

## Abstract

Recent studies provide evidence that high-fat diets (HF) trigger both i) a deficit of reward responses linked to a decrease of mesolimbic dopaminergic activity, and ii) a disorganization of circadian feeding behavior that switch from a structured meal-based schedule to a continuous snacking, even during periods normally devoted to rest. This feeding pattern has been shown to be a cause of HF-induced overweight and obesity. Our hypothesis deals with the eventual link between the rewarding properties of food and the circadian distribution of meals. We have investigated the effect of circadian feeding pattern on reward circuits by means of the conditioned-place preference (CPP) paradigm and we have characterized the rewarding properties of natural (food) and artificial (cocaine) reinforcers both in free-feeding *ad libitum* HF mice and in HF animals submitted to a re-organized feeding schedule based on the standard feeding behavior displayed by mice feeding normal chow (“forced synchronization”). We demonstrate that i) *ad libitum* HF diet attenuates cocaine and food reward in the CPP protocol, and ii) forced synchronization of feeding prevents this reward deficit. Our study provides further evidence that the rewarding impact of food with low palatability is diminished in mice exposed to a high-fat diet and strongly suggest that the decreased sensitivity to chow as a positive reinforcer triggers a disorganized feeding pattern which might account for metabolic disorders leading to obesity.

## Introduction

Feeding behavior is driven by energy demands, termed “homeostatic” feeding, and also by the hedonic gratification associated with eating a preferred food, referred to as “non-homeostatic” feeding [1]. The hedonic value of food is thought to contribute to its reinforcing properties [2]; [3]. Moreover, eating a preferred food has been shown to serve as a tool to manage anxiety and/or stress [4]. Thus, it can be stated that feeding behavior deals with the combination of energy balance status and neuropsychological components.

The influence of energy balance in regulating the activity of reward circuits is a question that remains mostly unanswered. Adiposity positively correlates with the preference for high-fat diets (HF) in obese individuals [5], [6] and excessive consumption of palatable food leads to compulsive-like eating [7]. Nevertheless other studies provide evidence that rats exposed to HF exhibit attenuated behaviors in response to both palatable food and amphetamine [8].

The activation of the reward system by food [9], [10] and recreational drugs [11], [12] suggests that common neural mechanisms underlie the reinforcing value of both food and drugs. Relevant to this, recent human studies reveal that obese individuals display decreased propensity to engage in the use of recreational drugs and a decreased prevalence of substance abuse disorders [13], [14], which is consistent with the hypothesis that obesity alters neural processing of rewarding stimuli.

The adipocyte-derived hormone leptin is a main candidate to coordinate energy balance and neuropsychological elements integral to food intake regulation [15]. In humans with congenital leptin deficiency, the activity of reward circuits increases in response to images of food and this is abolished by leptin therapy [16]. Other studies provide evidence that leptin reduces conditioned-place preference (CPP) for sucrose [17] or HF [18].

All these antecedents suggest that food intake behavior is driven by the balance/imbalance between the rewarding properties of food and the inhibition of feeding behavior triggered by feed-back mechanisms involving adipocyte-derived mediators, such as leptin. Nevertheless the way that feeding behavior influence food reward remains poorly understood. Therefore, we have investigated the influence of feeding behavior on the functionality of reward pathways. To design the study we have taken advantage of the fact that free-feeding mice undergoing HF treatment display an altered circadian pattern of meals distribution [19]. Under these conditions HF individuals gain more weight than their control lean counterparts, and also develop hyperleptinemia [20]. Moreover, a recent study carried out in our laboratory has evidenced that HF mice forced to adhere a standard pattern of feeding normalize body weight and adiposity [21]. The link between circadian processes and obesity has been an issue of research by other groups and us. Namely, initial investigation by Mistlberger et al. [22], [23] have evidenced that excessive diurnal feeding adversely contributes to body weight regulation in genetically obese rats. Otherwise we have recently reported that spontaneous disorganization of circadian feeding behavior triggered by HF diets is a cause of overweight/obesity in mice [24]. In the current study we have investigated the effect of circadian feeding pattern on reward circuits. Conditioned-place preference (CPP) paradigm was used to characterize the rewarding properties of natural (food) and artificial (cocaine) reinforcers both in *ad libitum* HF mice and in HF animals submitted to an organized feeding schedule.

## Materials and Methods

### Diet and feeding schedules

Four-week old male C57BL/6J mice (Harlan, Spain) weighing 16–18 g were housed under a 12 h light/12 h dark cycle, in a temperature-controlled room (22°C) with food and water *ad libitum.* The investigation conforms to the *Guide for the Care and Use of Laboratory Animals* published by the US National Institute of Health (NIH publication No. 85-23, revised 1996) and it was approved by the Ethics Committee of the San Pablo-CEU University (SAF2009-09714).

Animals were divided into two groups with similar average BW and assigned either to a control or to a high-fat diet (HF). Control (D12450B, 10 kcal % fat, 70 kcal % carbohydrates and 20 kcal % protein; 3.85 kcal/g) or high-fat (D12451, 45 kcal % fat, 35 kcal % carbohydrates and 20 kcal % protein; 4.73 kcal/g) diets were supplied by Test Diet Limited BCM IPS Ltd (UK). Both nocturnal (6 *pm*–8 *am*) and diurnal (8 *am*–6 *pm*) food intake were monitored during 28 days. After this period, both control and HF groups were divided in two subgroups. A subgroup of control mice was assigned to an *ad libitum* control treatment and will be referred as control-*al*. The other control subgroup received either at 8 *am* and 6 *pm* an amount of pre-weighed food identical to that consumed by the control-*al* subgroup between 8 *am*–6 *pm* and 6 *pm*–8 *am*, respectively. This subgroup will be referred as control-pair fed (control-*pf*). A subgroup of HF mice was also assigned to an *ad libitum* HF (HF-*al*) schedule and the other HF subgroup received both a nocturnal (6 *pm*) and a diurnal (8 *am*) meal of pre-weighed HF (forced-synchronization of food intake). In this case, chow portions were calculated in basis to the relative distribution of caloric intake detected in the HF-*al* group. Briefly, mice of this group consumed daily the same amount of calories than the corresponding matched HF-*al* mice but with a nocturnal/diurnal pattern identical to that detected in the control-*al* group. This group will be referred as HF-pair fed (HF-*pf*). This protocol was maintained during 28 additional days. Body weight was monitored twice a week during treatment. The last day animals were killed by decapitation, blood collected in chilled EDTA-coated polypropylene tubes and tissues dissected, weighed and frozen in liquid nitrogen. Groups of six animals of each treatment were killed at three-hour intervals, starting at 9 *am*. Animals were killed between 10–11 *am*.

### Chronic leptin administration

Ten-week old male mice were randomly assigned to groups receiving either subcutaneous leptin (0.1 mg/kg/12 h) or saline. Leptin (Sigma, USA) was given sc at a final concentration of 0.01 mg/ml in saline. Subcutaneous injections were performed at 10 *am* and 5 *pm* during 12 days, starting 7 days before place conditioning experiments.

### Place conditioning with cocaine

Place conditioning was carried as previously described [25] in a plexiglas apparatus formed by two compartments of the same size (20 cm length, 10 cm width, 15 cm height). One compartment had black floor and walls and the other was white. During cocaine and saline-paired sessions compartments remained closed by a removable guillotine door. The procedure selected for the current study consisted of a 5-day schedule with three phases (preconditioning, conditioning and testing). During preconditioning, animals were free to explore the two compartments for a 30-min period and behavior was monitored to calculate the time spent in each compartment. Conditioning phase consisted of a 3-day schedule of double conditioning sessions (*am* and *pm*), separated by a delay of at least 3 h. Mice receiving an ip injection of saline were immediately confined to the black compartment (30 min) on *pm* sessions (days 2 and 4) and on the *am* session on day 3. Cocaine (1–8 mg/kg) was administered before animals were confined to the white compartment on complementary sessions. For testing (day 5) mice were allowed to freely move throughout the apparatus and time spent in each compartment recorded. A biased approach was used and animals received the drug in the less preferred compartment identified in the preconditioning test, that was always the white compartment. This method has been shown to produce reliable CPP responses comparable with other experimental designs [26].

For forced-synchronization of food intake and chronic leptin experiments, 2 mg/kg of cocaine was used to induce conditioned place preference.

Results were expressed as % total time spent in the white compartment and compared to the time spent during preconditioning.

### Place conditioning with food

Place conditioning was carried out in the mentioned above apparatus and following a similar protocol, where cocaine administration was substituted by food availability in the white compartment. During preconditioning, animals freely explored the two compartments for a 30-min period and behavior was monitored to calculate the time spent in each compartment. During training mice were confined (30 min) to the white (baited) compartment during the morning (days 2 and 4) and afternoon sessions (day 3), and to the black compartment during the other training sessions. The white compartment was baited either with chocolate krispies (Kellogs, USA) or neutral non-caloric chow (Bio-Serv, USA). In order to avoid mnemonic effects of chow, free feeding was allowed after 1 h post-training. For testing (day 5) mice had free access to the whole apparatus for 30 min and the time spent in each compartment was recorded. Animals were food-restricted one week before preconditioning and during the CPP protocol to reach a 95% initial body weight. Before preconditioning, food was presented in home cages to habituate mice to chocolate/neutral food. A biased approach was used in these assays.

### Passive Avoidance Test (PAT)

The passive avoidance task was used to assess simple non-spatial learning ability [27], [28]. The passive avoidance apparatus consisted of two separate chambers (30×30×20 cm height): one light or white compartment and another dark or black. Each chamber was separated by a small guillotine door (3.5×5 cm) and grids were attached on the floor in the dark chamber (Ugo Basile, Mod. 7552). The test consisted of two phases, 1) Acquisition test: One hour before training, animals were transferred to the experimental room. Each animal was placed in the light chamber and the door to the dark compartment was opened after 10 s. Immediately after the animal entered the dark compartment, the door was locked and an electrical stimulation (0.36 mA) was applied for 2 sec. The time of latency for entering the dark compartment was recorded. Animals with a latency of more than 100 s were omitted from this research. 2) Retention test: Retention tests were performed to assess long-term memory 24 h after training. The animals were placed in the light chamber and after 10 s the door was opened. The time spent in this chamber before entering the dark chamber was measured. The latency in the retention session was expressed graphically and used in data analysis. In this phase, the foot shock was omitted. Cut-off time was set at 240s.

### Determination of plasma leptin and adiposity

After behavioral assays animals were killed by decapitation and plasma leptin concentration determined by specific radioimmunoassay (RIA) (Linco Research, USA; 4.9% intra-assay variation, 3.3% inter-assay variation). Lumbar, mesenteric, subcutaneous and epidydymal adipose tissues were dissected and weighed.

### Statistics

For body weight (BW), adipose tissues and circulating leptin values are expressed as means±S.E.M. Effects were analyzed by one-way ANOVA followed by the Newman-Keuls *post hoc* test. For CPP and passive avoidance experiments, effects were analyzed by two-way repeated measures ANOVA followed by the Bonferroni or Fischer's *post hoc* tests. *Diet* (HF or control) and *treatment* (cocaine or food) were considered as factors for two-way ANOVA. Statistical significance was set at p<0.05.

## Results

### Effect of diet and forced synchronization of feeding on body weight, adiposity and plasma leptin

**Figure 1:**
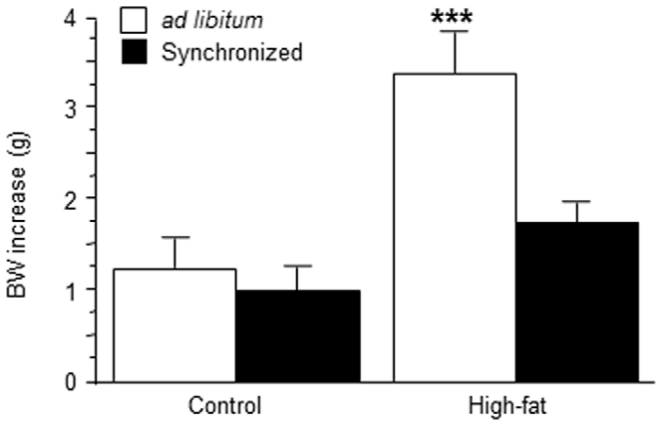
Effect of forced synchronization of feeding on body weight gain. Four-week old mice had access either to control or to high-fat diet during four weeks. After this period, animals were submitted to a forced synchronization protocol during four supplementary weeks. At the end point, body weight increase during the last four weeks was higher in high-fat *ad libitum* animals than in the other three groups. ***p<0.001, compared to the other groups (Newman-Keuls' test).

Body weight (BW), adipose tissues and circulating leptin were compared in animals fed on HF or control diets either with free (*al* groups) or controlled (*pf* groups) food access. As summarized in Table 1, *HF-al* mice exhibited heavier adipose tissue pads (p<0.001) than controls. An increase of plasma leptin concentration was also detected in these animals (p<0.001). Plasma leptin concentration was similar between control*-al* and *HF-pf* individuals (p<0.001). Regarding BW, two-way ANOVA revealed significant effects of both dietary treatment with HF (F_(1,34)_ = 21,520; p<0.001) and forced synchronization (F_(1,34)_ = 9,000; p<0.01) as well as a significant interaction between them (F_(1,34)_ = 4,856; p<0.05), which indicates that HF mice submitted to a forced synchronization protocol (*HF-pf*) displayed BW significantly smaller (p<0.001) than free-feeding HF animals (*HF-al*) (Figure 1).

**Table 1:** Body weight, plasma leptin and weight of adipose tissues.

	Control *ad libitum*	HF *ad libitum*	Control pair fed	HF pair fed
**Body weight (g)**	25.86±0.37	33.06±1.94**	25.63±0.47	27.65±0.60##
**Plasma leptin (ng/ml)**	5.40±0.30	16.00±1.7***	6.30±0.40	6.52±0.25
**MAT (mg/mm)**	10.19±1.13	22.94±3.16**	14.28±1.11	17.36±0.49#
**LAT (mg/mm)**	10.00±1.77	34.69±2.61***	7.75±0.95	11.07±1.15###
**SbAT (mg/mm)**	15.31±1.67	41.86±4.48***	18.90±2.45	28.59±4.04#
**ApAT (mg/mm)**	25.94±1.94	78.75±8.1***	16.25±2.60	24.40±4.55###

**Data are mean ± S.E.M. of 8–10 individual values. MAT (mesenteric adipose tissue), LAT (lumbar adipose tissue), SbAT (Subcutaneous adipose tissue), EpAT (epidimal adipose tissue);**

* **p<0.05;**

** **p<0.01;**

*** **p<0.001,** ***HF ad libitum vs*** **the three other groups;**

# **p<0.05,**

## **p<0.01,**

### **p<0.001,** ***HF pair fed vs HF ad libitum*** **. Tissue weights are expressed in mg** ***per*** **mm tibia length.**

### Dietary treatment with a high-fat diet reduces cocaine conditioned-place preference

**Figure 2:**
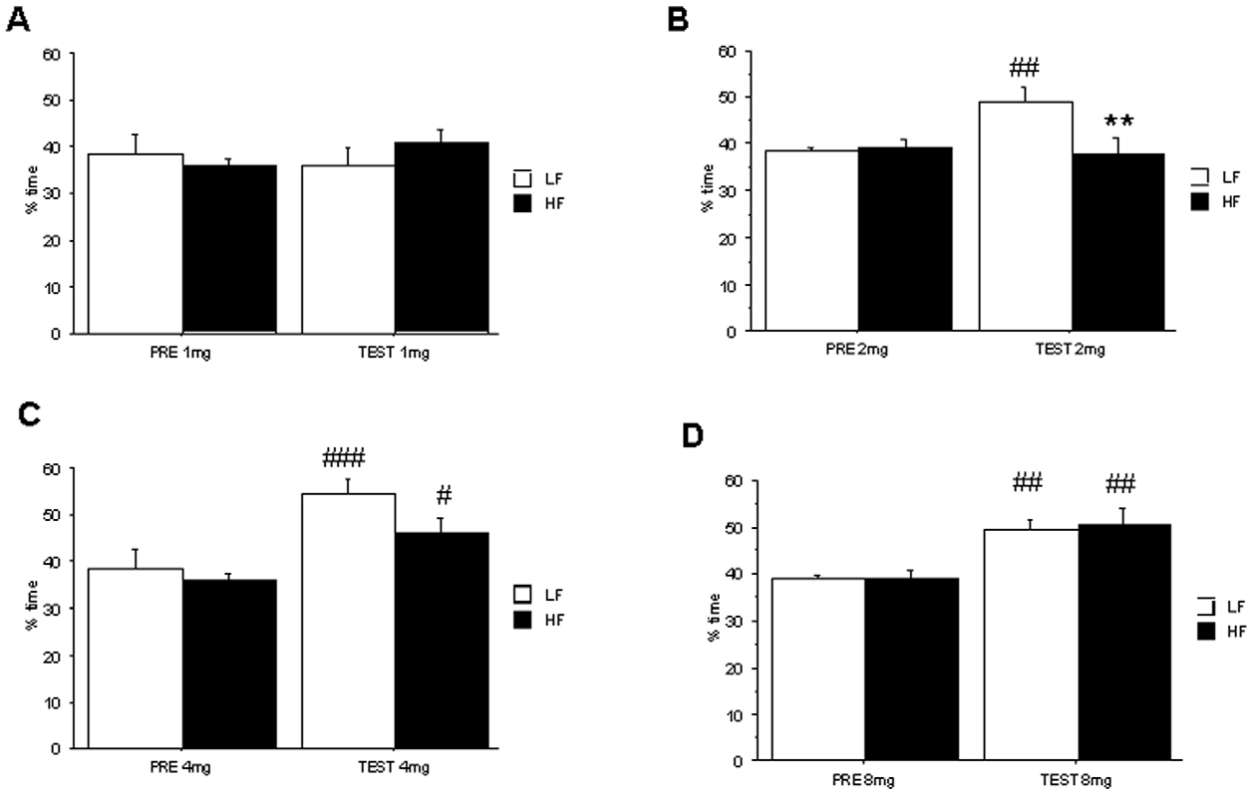
Conditioned-place preference induced by cocaine in control and HF C57BL/6J mice. CPP was induced by a three-day schedule treatment with 1 (**A**), 2 (**B**), 4 (**C**) or 8 mg/kg (**D**). Animals were tested during 30 min and results are expressed as % time spent in the white drug-paired arm. Values are mean±S.E.M of 7–8 animals (**p<0.01 for comparison between control and HF groups, and #p<0.05, ##p<0.01, ###p<0.001 for comparison between testing –TEST- and preconditioning –PRE- phases).

To evaluate the effect of HF diets on the perception of cocaine as a positive reinforcer, we compared CPP induced by the drug in control and HF individuals. As illustrated in Figure 2, 1 mg/kg cocaine failed to induce CPP (Figure 2A). In contrast, time spent in cocaine-paired compartment was higher during the testing (TEST) phase than during preconditioning (PRE) in control and HF animals receiving 4 mg/kg (F_1,14_ = 19.01, p<0.001, Figure 2C) or 8 mg/kg cocaine (F_1,13_ = 35,00 p<0.001, Figure 2D). At intermediate doses (2 mg/kg), cocaine induced CPP only in control mice (Figure 2B). Two-way ANOVA showed statistical differences for the interaction *“treatment x diet”* in 2 mg/kg cocaine animals (F_1,14_ = 5.80, p<0.01). Post-hoc analysis showed statistical differences between PRE and TEST phases in control animals (p<0.01) and between control and HF animals during TEST (p<0.01).

### Dietary treatment with high-fat diets reduces food reward in the conditioned-place preference

**Figure 3:**
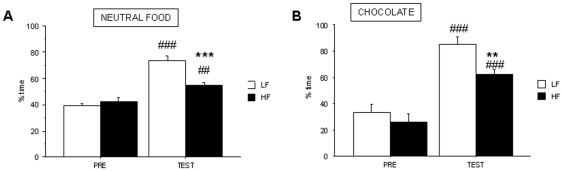
Conditioned-place preference induced by food in control and HF C57BL/6J mice. CPP was induced by a three-day schedule treatment with neutral food (**A**) or chocolate (**B**) pellets. Animals were tested during 30 min and results are expressed as % time spent in the white, food-paired, arm. Values are mean±S.E.M. of 12 animals for neutral food and 6–8 animals for chocolate (**p<0.01 for comparison between control and HF groups, and ##p<0.01, ###p<0.001 for comparison between testing –TEST- and preconditioning –PRE- phases).

To evaluate the effect of HF diets on the perception of food as a positive reinforcer, we compared CPP induced by non-caloric neutral food (Figure 3A) and chocolate krispies (Figure 3B) in control and HF mice. When neutral food was used, both control and HF mice spent more time in the food-paired compartment after conditioning. Two-way analysis showed statistical differences for *treatment* (F_1,22_ = 73.46, p<0.001) and the interaction *“treatment x diet”* (F_1,22_ = 17.23, p<0.001). Post-hoc analysis showed an effect significantly more robust in control than in HF mice and statistical differences between both groups during TEST phase (p<0.001). A similar result was found when chocolate flavoured food was used for conditioning (F_1,11_ = 126.04, p<0.001 for *treatment* effect) indicating chocolate krispies are positive reinforcers for both groups. Although both HF and control mice were also food-conditioned (Figure 3B), post-hoc analysis showed statistical differences in chocolate food-conditioning between control and HF animals during TEST phase (p<0.01).

#### Effects of HF diet in passive avoidance protocol

**Figure 4:**
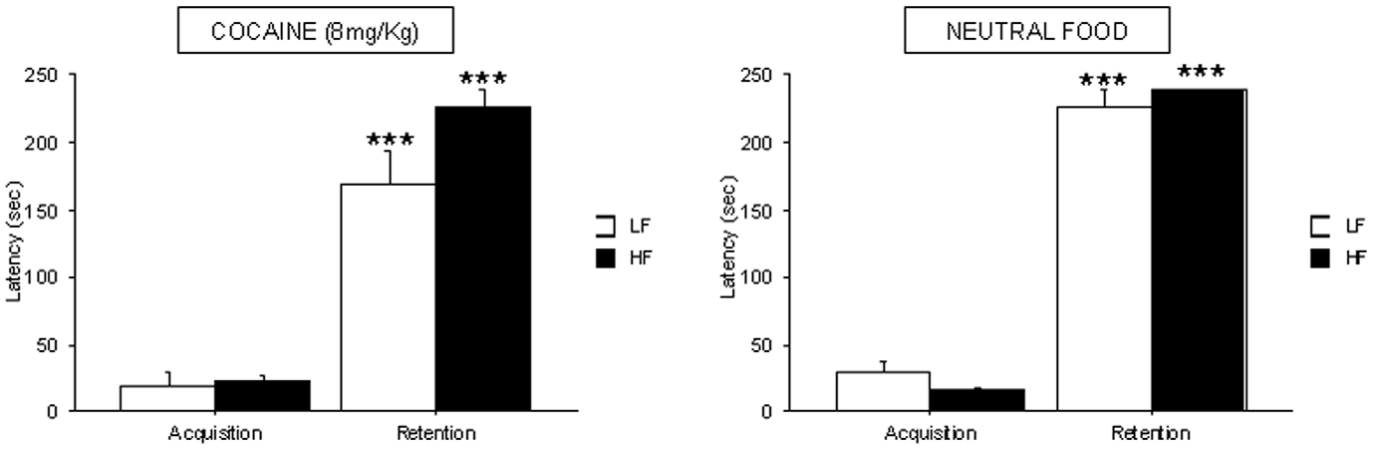
Passive avoidance. Latencies to enter the light compartment were measured both in control and HF mice 24 h after electrical shock. Cocaine induced CPP (8 mg/kg) did not modify latencies during the acquisition trial neither during the retention test (n = 5 for control and n = 7 for HF). Identical results were observed in animals after neutral food induced CPP (n = 8 for control and n = 9 for HF). Statistical differences were found between acquisition trial and retention tests indicating the absence of memory impairments in all groups (^***^p<0.001).

To confirm that differences in CPP between HF and control animals were not due to severe memory impairment, mice were submitted to a passive avoidance protocol after CPP induced either by cocaine (8 mg/kg) or neutral food. Series As illustrated in Figure 4 no statistical differences were found in the retention latency between control and HF treated animals when passive avoidance protocol was carried out after cocaine induced CPP or neutral food CPP. Therefore, treatments did not affect the acquisition and consolidation phases of this memory process.

#### Effect of forced synchronization of feeding behavior on cocaine- and food-induced conditioned place preference

**Figure 5:**
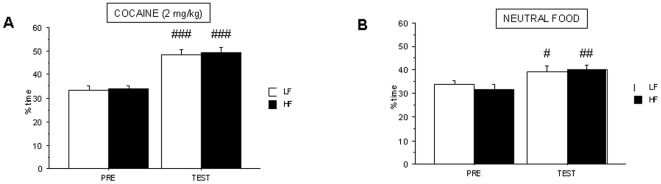
Forced synchronized diet in cocaine 2 mg/kg CPP and food CPP. CPP was induced after re-organization of feeding behavior in control and HF groups. Animals were tested during 30 min and results are expressed as % time spent in the white food (A)or cocaine-paired (B) arm. Values are mean±S.E.M. of 15–16 animals #p<0.05, ##p<0.01, ###p<0.001 for comparison between testing –TEST- and preconditioning –PRE- phases).

To evaluate the influence of forced synchronization of feeding behavior on reward, we characterized CPP induced either by cocaine (2 mg/kg) or by neutral non-caloric food in control and HF mice undergoing a controlled schedule of food supply (control*-pf* and *HF-pf*, respectively). As illustrated in Figure 5A, CPP was triggered by 2 mg/kg of cocaine both in control*-pf* and *HF-pf* individuals (F_1,32_ = 54.97, p<0.001) and no statistical differences were found between both groups during TEST phase. Figure 5B shows the effect of neutral food on CPP. In this case, two-way ANOVA also revealed an effect of neutral food on control*-pf* and *HF-pf* (F_1,29_ = 14.32, p<0.001) mice. No differences were found between control*-pf* and *HF-pf* animals. These results indicate that reorganization of feeding behavior in HF mice (*HF-pf*) abolish the effects observed in free-feeding HF individuals (*HF-al*).

### Effect of exogenous leptin on cocaine-induced conditioned-place preference

Mice receiving leptin treatment (0.1 mg/kg/12 h) were compared to age-matched controls by using the cocaine CPP protocol. Leptin treatment led to plasma leptin concentrations ranging between 21 ng/ml (2 h after leptin administration) and 7 ng/ml (detected immediately before the next administration).

**Figure 6:**
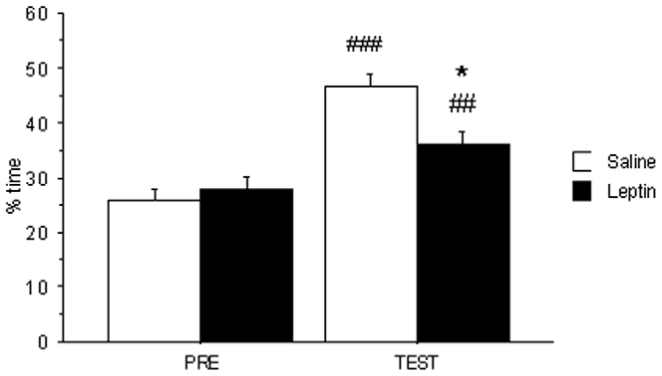
Chronic leptin administration impairs cocaine 2 mg/kg CPP. Cocaine CPP was induced after chronic leptin (0.1 mg/kg/12 h/12days) administration in control and HF groups. Animals were tested during 30 min and results are expressed as % time spent in the white cocaine-paired arm. Values are mean±S.E.M of 11–12 animals (*p<0.05 for comparison between control and HF groups, and ##p<0.01, ^###^p<0.001 for comparison between testing –TEST- and preconditioning –PRE- phases).

As illustrated in Figure 6, cocaine (2 mg/kg) induced CPP in control and leptin-treated animals (F_1,21_ = 38.65, p<0.001 for the *treatment* effect and F_1,21_ = 7.28, p<0.05 for the interaction *“treatment x diet”*). Nevertheless, post-hoc analysis showed statistical differences between both groups in the time spent in cocaine-paired compartment in TEST phase (p<0.001). This result showed that exogenous leptin has a similar effect on reward than diet-induced obesity (DIO) evoked by HF diets.

## Discussion

Previous studies have evidenced that obesity induced by HF diets in mice is linked to a disordered feeding pattern [29]; [30]. In the current study we demonstrate that HF diets diminish food and cocaine reward in mice and this behavioral impairment is not observed in animals forced to adhere to a standard meal-based feeding behavior (“forced-synchronization”). The eventual influence of stress, due the time-constrained access to food during the forced-synchronization protocol, as a source of behavioral changes can be ruled out because plasma corticosterone levels were unaffected by free-feeding HF [24] and also by forced synchronization (HF-*al* 375.0±42.9 nmol/l *vs* 427.3±18.6 nmol/l in HF-pf. Values obtained at 1000 am).

A main result of this study concerns the negative impact of diet-induced obesity (DIO) on food and cocaine reward, which would suggest that free-feeding HF diets trigger the inhibition of dopaminergic mesolimbic pathways involved in reward [8]. Nevertheless, our data provide evidence that diet composition is not pivotal in driving the activity of reward pathways. Because feeding reorganization tends to normalize BW and adiposity ([31]; this study), we suggest that signals derived from the adipose tissue might account for reward inhibition associated to DIO. Otherwise, our results strongly suggest that food, considered as a positive reinforcer, would trigger feed-back mechanisms recruiting physiological markers of energy status, which in turn would limit food reward. In this sense, we propose leptin as the main candidate to connect adiposity and motivation for food. In fact, mesolimbic dopaminergic neurons are sensitive to leptin [32] and leptin infusion into the ventral tegmental area (VTA) has been shown to suppress food intake and firing of these neurons in rats [33]. As proposed by Cota et al. [15], we hypothesize that motivational aspects of feeding behavior concern an adipose tissue-mesolimbic axis. The pivotal role of leptin is stressed by results showing that control mice receiving a chronic treatment with leptin display poor motivation for cocaine or food reward.

Interestingly, these animals were hyperleptinemic but exhibited normal BW. Leptin posology (0.1 mg/kg/12 h) was chosen to induce mild hyperleptinemia without causing BW loss (data not shown). Under our conditions plasma leptin concentration ranged between 21 ng/ml, measured 2 h after leptin administration, and 7 ng/ml, determined before the next dose. This is a very important detail because other authors have reported inhibitory effects of leptin on dopaminergic activity and BW after stereotaxic administration into the VTA of doses as high as 1 µg/animal in rats [33]. Our current results demonstrate that exogenous leptin, which yields mild hyperleptinemia in the range of DIO-evoked hyperleptinemia, is enough to promote reward inhibition. Nevertheless the involvement of leptin in the inhibition of food and/or cocaine-reward, although strongly suggested, cannot be concluded and other studies aimed at blocking leptin receptor signaling in HF mice should be performed.

Our study raises the hypothesis of a sequential connection between disorganized feeding behavior, reward deficits and obesity. As previously reported, forced synchronization of feeding prevents gain of BW by HF diets [31]. In the current study we show that forced synchronization also prevents reward impairment. The question that emerges from our study deals with the link between lack of food reward and obesity. This is a pivotal issue in this study because HF diets act as positive reinforcers [34] rather than as aversive stimuli. Thus, we propose that the initial enhancement of reward by HF diets motivate animals to eat more than they need to fulfill their energetic needs. In fact, animals on HF initially display hyperphagia [20]. Overeating leads to a rapid enlargement of adipose pads and to a subsequent increase of leptin production [20]; [35]. At this time-point, hyperleptinemia would counterbalance the rewarding properties of food and other reinforcers, finally leading to an altered feeding behavior schedule, which seems to account for overweight/obesity [31].

The link between circadian rhythm and changes in motivation for food and cocaine has been the issue of previous research and it seems that abnormal circadian rhythms can facilitate drug addiction and vice versa [36], [37].These events seem to be connected by the so called clock genes [38]. Interestingly we have seen in a previous study that HF up-regulates the clock gene Per2 within the hypothalamus and white adipose tissue. Per2 expression appears to be normalized after forced synchronization of feeding behavior [24].

In the current study we propose that shift or circadian feeding behavior trigger a (partial) loss of motivation for food that is reversed by forced synchronization of feeding behavior. We have no data regarding Per2 expression in areas involved in volitional consumption of food but it can be speculated that Per2 expression would be increased in these reward-related areas, as occurs in the hypothalamus and in adipose tissue. In fact, Per2 mutations have been shown to be related with strong cocaine-induced CPP [39], [40].

Our investigation presents important differences with previous studies in this field because it is the first study aimed at demonstrating the link between circadian feeding behavior and food reward. Data presented here allow us to speculate the possible connection between reward inhibition and obesity through a vicious circle integrated by i) initial increase of BW triggered by non-homeostatic feeding, ii) hyperleptinemia, iii) inhibition of food reward, iv) disordered feeding, and v) metabolic alterations triggering adiposity increase and hyperleptinemia.

In summary, the current study provides evidence of an alteration of motivational behavior pattern on mice undergoing a dietary treatment with a HF diet. We propose that free-feeding with highly palatable food together with a dysfunction of neural reward pathways is a condition leading to an increase of caloric efficiency and further obesity.
